# Laparoscopic Gastric Jejunal Bypass for Pyloric Stenosis After Corrosive Gastritis in an Adult: A Case Report

**DOI:** 10.7759/cureus.93066

**Published:** 2025-09-23

**Authors:** Rintaro Kawahara, Nobuhiro Nakazawa, Akihiko Sano, Makoto Sakai, Hiroshi Saeki

**Affiliations:** 1 General Surgical Science, Gunma University, Maebashi, JPN

**Keywords:** corrosive gastritis, gastric jejunal bypass, gastric outlet obstruction, laparoscopy, pyloric stenosis

## Abstract

Corrosive gastritis in adults is caused by the ingestion of corrosive substances and can present with various symptoms. In severe cases, it may result in resorptive gastritis leading to pyloric stenosis. While oral gastric mucoprotective agents and endoscopic balloon dilation are used to treat pyloric stenosis, surgery is required in many cases. We report a case of pyloric stenosis secondary to corrosive gastritis, successfully treated with laparoscopic gastric jejunal bypass. A 64-year-old Japanese female with schizophrenia attempted suicide by ingesting a caustic alkaline solution, resulting in corrosive gastritis and subsequent pyloric stenosis. In our patient, the stenosis measured approximately 4 cm in length and was further complicated by additional factors, rendering endoscopic treatment unsuitable. Therefore, we performed a laparoscopic gastric jejunal bypass. This case demonstrates that laparoscopic gastric jejunal bypass is a safe and effective option for pyloric stenosis secondary to corrosive gastritis. It may serve as a feasible and less invasive surgical option, especially for patients with psychiatric comorbidities or high operative risk.

## Introduction

Corrosive gastritis is caused by the ingestion of caustic substances and can lead to severe tissue injury. Such ingestion may result in esophagitis and gastritis, with a spectrum of gastrointestinal damage ranging from mild mucosal erosion to ulceration, perforation, and scar formation. In severe cases, corrosive gastritis may lead to pyloric stenosis, which occurs in approximately 5% of cases and often requires surgical intervention. Although a few reports have suggested that oral gastric mucoprotective agents, systemic steroids, or endoscopic bougie dilation may be effective in selected cases [1-3], surgery is generally performed when these therapies are ineffective. It has generally been believed that gastric injury from alkaline substances is relatively rare, as gastric acid neutralizes the caustic effect of alkali within the stomach [4]. In the present case, however, pyloric stenosis developed as a consequence of corrosive gastritis caused by ingestion of an alkaline solution. We report a case of laparoscopic gastric jejunal bypass performed for pyloric stenosis secondary to corrosive gastritis.

## Case presentation

A 64-year-old Japanese female with a history of schizophrenia attempted suicide by ingesting 1,200 mL of sodium hypochlorite solution (®Haiter). She was initially admitted to a psychiatric hospital for observation.

One month later, due to worsening psychiatric symptoms, she was transferred to our institution. Conservative management was initiated for mild esophagitis and corrosive gastritis, and total parenteral nutrition was started. However, two months after ingestion, she developed progressive gastric outlet obstruction due to pyloric stenosis secondary to corrosive gastritis. Her symptoms were characterized by postprandial nausea and recurrent vomiting, particularly after soft meals. She was referred to our department for further evaluation, as the stenosis had resulted in impaired gastric emptying.

**Figure 1:**
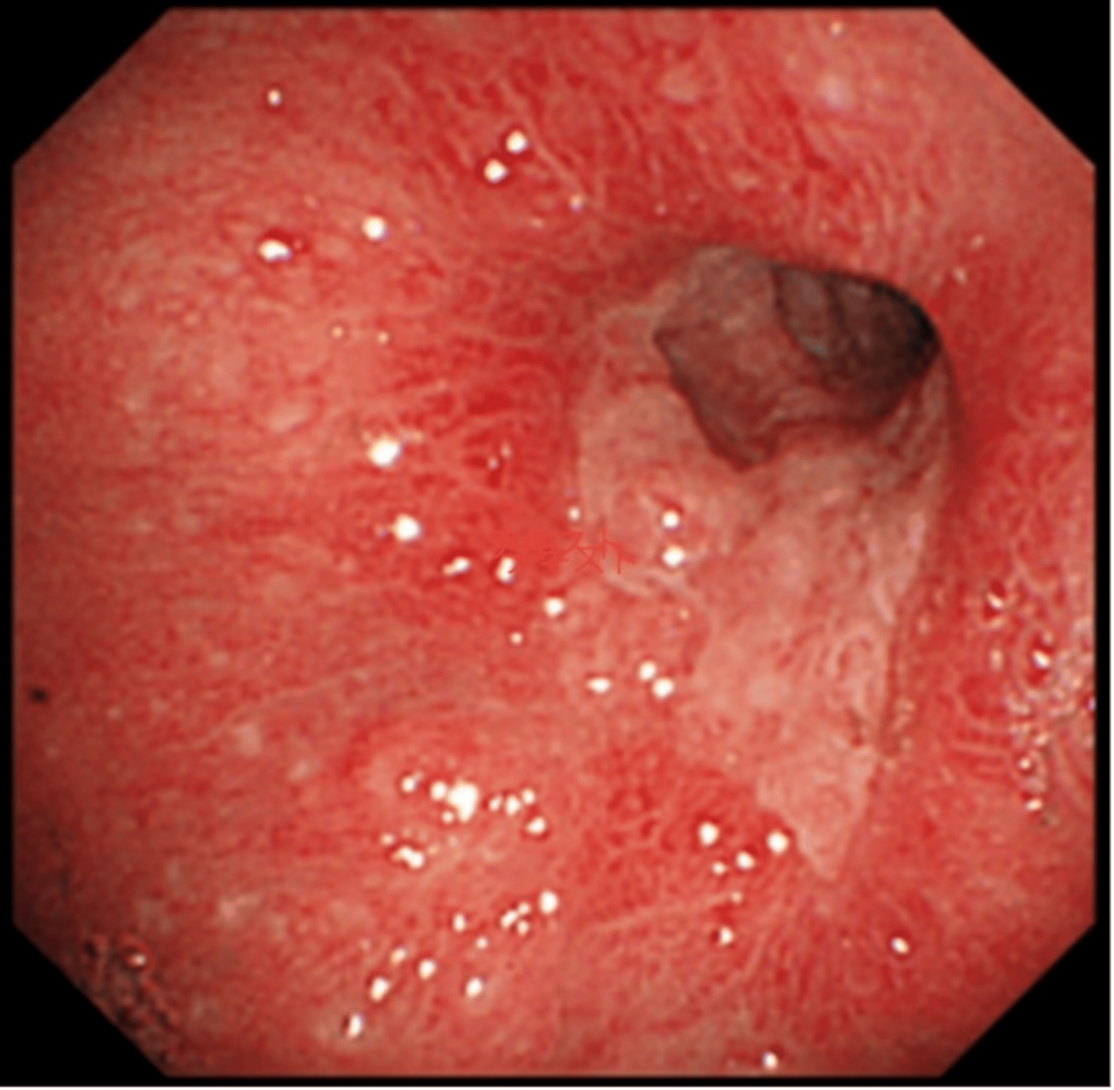
Upper gastrointestinal endoscopy showing severe gastropyloric stenosis.

**Figure 2:**
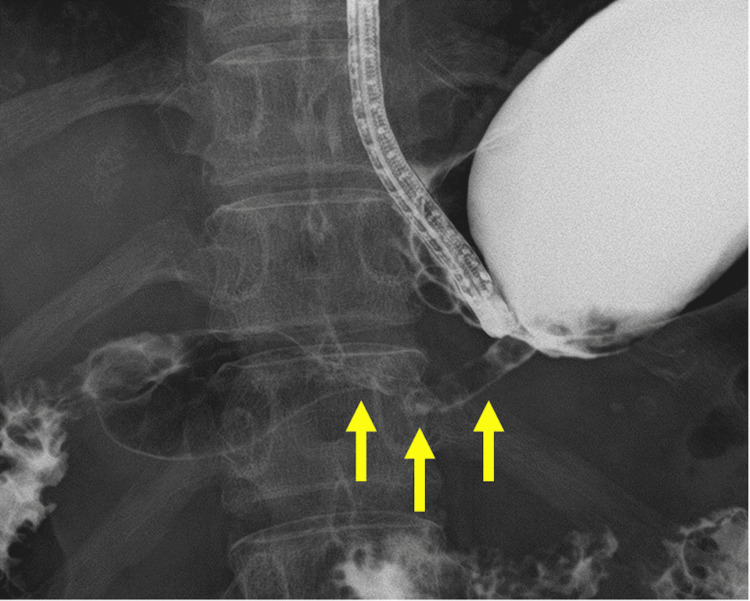
Gastrofluoroscopy and gastropyloric stenosis.

The blood test results showed an albumin level of 3.7 g/dL, a total lymphocyte count of 790/μL, and a Prognostic Nutritional Index (PNI) of 40.95 (Table 1). Upper gastrointestinal endoscopy revealed a stenotic segment in the pyloric antrum that prevented advancement of the endoscope (Figure 1). Biopsy of the stenotic lesion demonstrated inflammatory changes without evidence of malignancy. Gastrofluoroscopy confirmed a 4-cm-long stenosis at the pylorus, although contrast passage was still observed (Figure 2).

**Table 1:** Pre- and postoperative laboratory findings

Parameter	Preoperative value	Postoperative day 3	Postoperative day 10	Normal range	Unit
Hematocrit (Ht)	39.5	35.5	36.5	35.0–45.0	%
Hemoglobin (Hb)	13.2	11.5	11.9	11.8–15.1	g/dL
Platelets (Plt)	194	141	199	160–350	10³/μL
White blood cells (WBC)	3.4	3.4	3.0	4.0–9.6	10³/μL
Neutrophils	2.28	-	1.77	1.97–6.44	10³/μL
Lymphocytes	0.79	-	0.91	1.27–3.26	10³/μL
Eosinophils	0.02	-	0.06	0.03-0.50	10³/μL
Basophils	0.01	-	0.01	0.00–0.09	10³/μL
Monocytes	0.33	-	0.24	0.12–0.50	10³/μL
Total protein (TP)	6.8	5.2	5.8	6.3–7.9	g/dL
Albumin (Alb)	3.7	2.6	3.0	3.9–5.0	g/dL
Aspartate aminotransferase (AST)	24	14	31	13–33	U/L
Alanine aminotransferase (ALT)	39	18	72	6–27	U/L
Blood urea nitrogen (BUN)	17	12	11	8–20	mg/dL
Creatinine (Cre)	0.56	0.47	0.54	0.46–0.79	mg/dL
Sodium (Na)	141	143	144	137–145	mEq/L
Potassium (K)	3.9	3.5	4.3	3.5–4.8	mEq/L
Chloride (Cl)	107	108	107	100–107	mEq/L

Endoscopic balloon dilation was initially considered; however, due to the length of the stenosis and the associated risk of gastrointestinal perforation, the procedure was deemed unsuitable. Given her psychiatric comorbidity, overall physical status, and the need to minimize surgical invasiveness, we elected to perform a laparoscopic gastric jejunal bypass.

**Figure 3:**
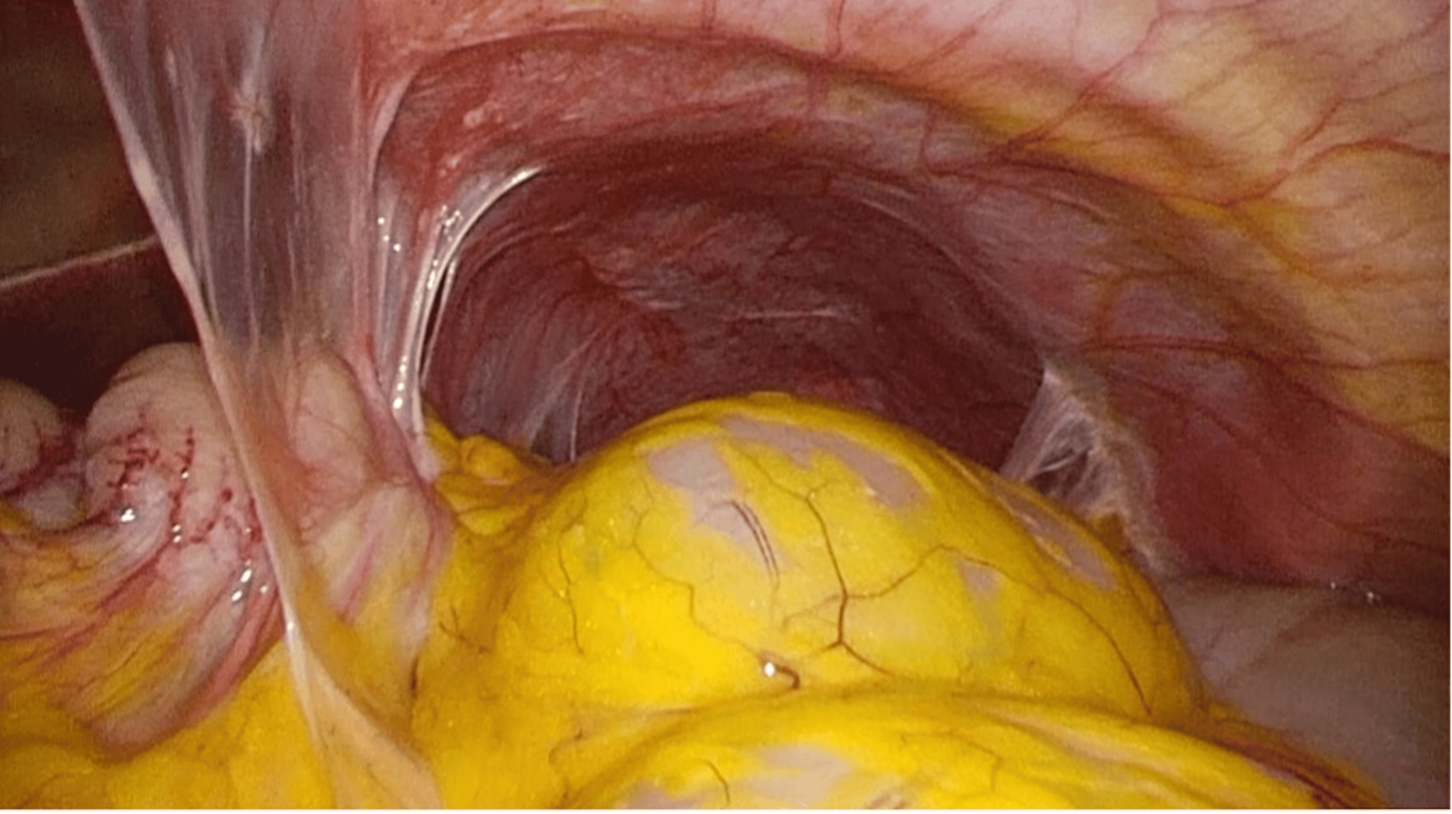
Laparoscopic findings: fibrous adhesions around the stomach.

The procedure was performed laparoscopically using four trocars inserted in the umbilical region, right epigastrium, right lower abdomen, and left epigastrium. Intraoperatively, severe fibrotic stenosis was observed in the pyloric region, accompanied by dense adhesions involving the surrounding tissues and a large surgical mesh, likely from a prior abdominal procedure (Figure 3). Careful adhesiolysis was undertaken to ensure an adequate operative field. The gastric lumen was evaluated endoscopically and confirmed to be unobstructed proximal to the stenosis. Based on the anatomical findings, an incision line was marked on the anterior wall of the gastric body, proximal to the lesion, to ensure optimal orientation and access for anastomosis. A side-to-side gastric jejunal anastomosis was created using an Endo GIA stapler (®Signia, Medtronic, Dublin, Ireland) in the jejunum, 25 cm distal to the ligament of Treitz. A Braun enteroenterostomy was then performed 5 cm distal to the gastrojejunostomy using the same stapler. All enterotomies were closed with 3-0 V-Loc (Medtronic) absorbable sutures, and Petersen’s defect was closed using 3-0 nonabsorbable V-Loc sutures (Medtronic).

The patient resumed oral intake with water on postoperative day (POD) one and progressed to solid food by day three. Her postoperative course was uneventful, and her nutritional intake was deemed satisfactory. She was transferred back to the psychiatric ward on POD seven. Laboratory data before and after surgery are summarized in Table 1. Compared with the preoperative values, the results on POD three and POD 10 showed normalization of electrolytes and improvement in nutritional and hematological parameters, indicating postoperative recovery and clinical benefit from the surgery. Following stabilization of both gastrointestinal and psychiatric symptoms, she was discharged from the hospital four months later.

## Discussion

Ingestion of corrosive substances is typically accidental in children, whereas in adults it usually occurs during suicide attempts. Symptoms in the acute phase following ingestion of a caustic substance include pain and burning sensations in the mouth and throat, retrosternal and epigastric pain, nausea, and vomiting. Gastroesophageal stenosis due to gastroesophagitis can develop in the chronic phase. The severity of gastroesophagitis is assessed by upper gastrointestinal endoscopy [1]. The mechanisms of corrosive gastroesophagitis differ between acidic and alkaline substances. Acidic substances cause coagulation necrosis on the mucosal surface and generally do not induce deep inflammation. In contrast, alkaline substances cause liquefaction necrosis, have higher surface tension, and remain in the tissue for a longer period. Therefore, inflammation caused by alkaline substances often penetrates more deeply into the tissues [5].

Endoscopic balloon dilation is the primary minimally invasive treatment for benign pyloric stenosis. Successful balloon dilations for pyloric stenosis following ingestion of caustic substances have also been reported [2]. However, in our patient, the stenosis measured over 2.5 cm in length and was therefore deemed unsuitable for this treatment. The stenosis measured 4 cm, which exceeds the generally accepted limit for endoscopic balloon dilation.

Currently, reports on surgeries for gastric outlet stenosis caused by ingestion of corrosive substances are limited and mostly describe open procedures. To date, approximately 80 cases of open surgeries for this condition have been reported [6-8]. Reports of minimally invasive surgery for corrosive gastritis-induced stenosis are limited. Kanyama et al. reported a case of severe corrosive gastritis successfully treated with laparoscopic distal gastrectomy [9], while Ayyaz et al. described laparoscopic gastrojejunostomy as an effective option in patients with stable general condition and comorbidities [10]. Although corrosive gastritis may be associated with an increased risk of gastric cancer, reported cases of gastric cancer developing after corrosive gastritis remain extremely limited [11]. Therefore, there is no clear evidence to support whether resection with reconstruction or gastrojejunostomy is the preferred surgical approach in such cases, and the relative superiority of either approach remains unclear. In the present case, laparoscopic gastric jejunal bypass was selected due to concerns about psychiatric exacerbation and the need to minimize surgical invasiveness.

Gastrojejunostomy can be performed on either the anterior or posterior wall of the stomach, and the optimal site remains controversial in terms of patient quality of life. With the widespread adoption of laparoscopic surgery in recent years, the anterior wall has more commonly been selected for gastrojejunostomy.

We expanded the discussion to clarify our rationale for performing a Braun anastomosis in addition to gastrojejunostomy. In this case, a Braun anastomosis was performed to divert bile and pancreatic secretions away from the gastric remnant, thereby reducing bile reflux and potentially lowering the risk of biliary gastritis and its associated symptoms [12]. Although the routine use of Braun anastomosis remains a matter of debate, we have adopted it selectively in cases where reducing reflux-related complications is a concern, as it may contribute to improved postoperative quality of life.

There are only a few reports of laparoscopic surgery for pyloric stenosis resulting from corrosive gastritis. Further reports and discussions are warranted to establish optimal treatment strategies.

This case report presents an early experience with laparoscopic gastrojejunostomy for pyloric stenosis following corrosive gastritis. The decision to pursue this approach was based on the patient’s underlying psychiatric condition, the reduced invasiveness of laparoscopic procedures compared to resections, and the length of the stenotic segment. The laparoscopic gastrojejunostomy was successfully completed, with the patient recovering promptly and experiencing no postoperative complications. This case demonstrates that laparoscopic gastrojejunostomy can be a safe and effective treatment option for pyloric stenosis secondary to corrosive gastritis. It may represent a feasible and less invasive surgical alternative, particularly for patients with psychiatric comorbidities or elevated operative risk.

## Conclusions

Laparoscopic gastric jejunal bypass is a safe and effective surgical treatment for pyloric stenosis secondary to corrosive gastritis. This minimally invasive approach offers particular advantages for patients with psychiatric comorbidities or those at high surgical risk. Further accumulation of cases is necessary to determine the optimal treatment strategy for this rare condition.
